# Efficacy of Dexrazoxane in Cardiac Protection in Pediatric Patients Treated With Anthracyclines

**DOI:** 10.7759/cureus.37308

**Published:** 2023-04-08

**Authors:** Parya Rahimi, Behsheed Barootkoob, Ahmed ElHashash, Arun Nair

**Affiliations:** 1 Medicine, First Faculty of Medicine, Charles University, Prague, CZE; 2 Pediatrics, Saint Peter’s University Hospital, Somerset, USA

**Keywords:** chemotherapy-related toxicity, pediatrics cardiotoxicity, dexrazoxane, anthracycline-induced cardiomyopathy, pediatric cancer

## Abstract

Cancer is one of the leading causes of morbidity and mortality in the pediatric population with the most common cancer being acute lymphoblastic leukemia. One of the most common drugs used in the treatment is the anthracycline group of chemotherapeutic agents, and a major side effect is cardiotoxicity. Dexrazoxane, a member of the cardioprotective agents' group of medications, is the only current FDA-approved medication to tackle cardiotoxicity. The mechanism of action in which dexrazoxane is cardioprotective is by halting necroptosis in cardiomyocytes after anthracycline therapy and concurrently binds with iron and reduces the formation of anthracycline-iron complexes and reactive oxygen species. The efficacy of dexrazoxane has been demonstrated in clinical trials within the pediatric population with roughly 60%-80% reduction in risk of developing cardiotoxicity with a very tolerable and limited side effect profile. Further research is required to not only establish the efficacy of dexrazoxane within the pediatric population but also to explore other medications that may serve alongside the function of dexrazoxane.

## Introduction and background

Cancer is a leading cause of morbidity and mortality in the pediatric population under the age of 19 with approximately 400,000 patients being diagnosed annually. The most commonly diagnosed cancer in the pediatric population is acute lymphoblastic leukemia (ALL) [1]. Fortunately, chemotherapeutic interventions have allowed for an increase in the survival of cancer patients from 50% to 80%, with a drawback being an increase in rates of premature heart disease among survivors [1]. Currently, the mainstay of treatment includes the use of anthracycline medications in more than 50% of childhood malignancies from which a prominent side effect that has been noted includes cardiotoxicity leading to a dose-dependent development of cardiac disease [1].

Anthracyclines provide therapeutic effects through multiple mechanisms, including inhibition of DNA synthesis, DNA binding with alkylation, and DNA cross-linking [2]. Another component of the mechanism of action includes the interference with helicase activity and separation of the DNA strands as well as the formation of free radicals and lipid peroxidation. Although the pathophysiology of cardiotoxicity with the use of anthracyclines is unclear, a potential theory suggests that the reactive oxidative species from anthracycline exposure may be the culprit [2]. Cardiotoxicity initially may be asymptomatic but can be identified on imaging modalities, such as echocardiograms, as a cardiac dysfunction or as a structural abnormality resembling dilated cardiomyopathy; this may eventually manifest into an irreversible restrictive pattern of cardiomyopathy [3]. Once patients are diagnosed with congestive heart failure (CHF) secondary to anthracycline exposure, the five-year survival rate reduces to 50% and it is imperative to note that children treated with anthracyclines tend to have a 5- to 15-fold increase in the risk of developing CHF compared to the general population [2]. As mentioned earlier, the dose-dependent manner in which anthracyclines are administered plays a role in the development of cardiotoxicity; as with a cumulative dose of more than 300 mg/m^2^, a notable increase in the risk of cardiotoxicity is noted; however, subclinical anomalies were noted on echocardiography with doses as low as 100 mg/m^2^ [3].

Risk factors associated with increased cardiotoxicity of anthracyclines can be categorized as therapy-related and modifiable and non-modifiable risk factors. Therapy-related risk factors include high cumulative doses used and concurrent use of radiation to the mediastinum or cranium. A possible association between cranial radiation and heart disease could be due to its effect on the hypothalamus and pituitary glands, which may result in a growth hormone deficiency [4]. One example of a non-modifiable factor includes the unique genetic makeup of individuals; mutations in multiple genes including *ABCC5*, *NOS3*, *HFE*, and *CBR3* have shown different susceptibility to the effect of anthracyclines [5]. It is interesting to note that gender also has an influence on susceptibility as studies have shown females to be more vulnerable to the side effects of anthracyclines compared to men, at the same cumulative dosages [3]. This may be due to the presence of a higher fat content in females that lead to poorer absorption of anthracyclines, as anthracyclines have poor fat absorption, and therefore resulting in a higher serum concentration [3]. Ethnicity may also play a role in different susceptibilities to developing cardiac manifestations as African-Americans have been shown to be at a higher risk compared to other races [3].

Currently, several methods to decrease the risk of heart failure can be implemented in adults with exposure to anthracyclines; however, there is not enough data to extrapolate to the pediatric population [2]. Two suggested strategies of cardioprotection in the pediatric population would be to either discontinue the administration of anthracycline medication alongside using cardiac remodeling medications or the use of dexrazoxane. Reducing or ceasing administration may not be ideal due to interference with chemotherapeutic efficacy, and a clinical decision regarding the benefits versus risks to the patient would have to be made. The use of cardiac remodeling agents such as angiotensin-converting enzyme (ACE) inhibitors or beta-blockers has not been fully studied in the pediatric population in the setting of persistent left ventricular systolic dysfunction (LVSD), and it is recommended that cancer survivors should have continued follow-ups with echocardiograms to monitor long-term effects of treatment [6]. The other strategy involves the use of dexrazoxane, and according to the current literature, when dexrazoxane has been given concomitantly with anthracycline therapy, both short- and mid-term cardiotoxicity risks have been reduced without compromising anti-tumor activity [7]. This literature review aims to compare and reassess the efficacy and toxicity of dexrazoxane as a cardioprotective treatment in the pediatric population being treated for leukemias using anthracyclines and also will discuss alternative methods of cardiac protection in patients exposed to anthracyclines with different regimens.

## Review

Current use of dexrazoxane

Dexrazoxane belongs to the pharmaceutical class of cardioprotective agents [8], which is structured as an enclosed ring and hydrophilic in nature, therefore, allowing it to diffuse into cells easily [9]. When metabolized, it forms a structure similar to ethylenediaminetetraacetic acid (EDTA), which is a potent iron chelator. It has been found to alter the path toward apoptosis and halt necroptosis in cardiomyocytes after therapy with doxorubicin and, simultaneously, binds with iron and reduces formations of anthracycline-iron complexes and therefore reduces the production of reactive oxygen species [9]. Dexrazoxane is also effective in reducing DNA damage, one of the mechanisms of action of anthracyclines, by inhibiting DNA topoisomerase II, thereby stimulating the selective degeneration of topoisomerase IIb, which is a molecular target of anthracyclines [9].

Currently, dexrazoxane is the only Food and Drug Administration (FDA)-approved medication used in the prevention of anthracycline-induced cardiotoxicity. Initially, the medication was approved in patients with metastatic breast cancer being treated with more than 300 mg/m^2^ of doxorubicin [10]; however, in 2014, it was approved for the prevention of anthracycline-induced cardiotoxicity in age groups 0-16 years [11]. The dosing regimen of dexrazoxane consists of a bolus infusion over 15 minutes immediately before starting anthracyclines, at a ratio of 10:1 to doxorubicin. To calculate the ratio of dexrazoxane with other anthracyclines, doxorubicin is used as control, resulting in a ratio of 5-10:1 with doxorubicin, 40:1 with mitoxantrone, and 50:1 with idarubicin [6]. Dosing adjustments need to be made in renally impaired patients with moderate to significant impairment. Adjustments also need to be made for both doxorubicin and dexrazoxane in patients with hepatic impairment while still maintaining a 10:1 ratio [9]. In humans, the half-life of dexrazoxane is approximately two hours. Less than 2% of dexrazoxane is bound to plasma proteins in blood [12].

The European Medical Agency (EMA) contraindicates dexrazoxane for the treatment of cardiotoxicity in children due to the claim of increased risk of secondary malignancy and unestablished efficacy of dexrazoxane in children [13]. In a prospective study regarding Hodgkin’s lymphoma, the use of dexrazoxane showed an increased incidence of myelodysplastic syndrome and other cancers [14]. However, various other studies demonstrated no association between the two groups, and hence, no differences in the occurrence of secondary malignancies in groups treated with dexrazoxane and those who were not. These discrepancies can be due to the initial study not taking into account cumulative doses of etoposide, cyclophosphamide, and doxorubicin or administration of granulocyte colony-stimulating factor (G-CSF) as confounding variables to the resulting increase in rates of secondary malignancies [14]. Factors that increased the hazard ratio of developing secondary malignancy included a history of radiation, higher total doses of etoposide, cyclophosphamide, and anthracyclines, longer therapy duration with anthracyclines, short anthracycline free interval, and longer total duration of chemotherapy.

Through multivariate analysis, studies showed a 1.05 times increase in malignancy for every one-month prolongation of anthracycline use as well as a decrease in the incidence of 0.99 times per month since the last administration of anthracyclines [14]. In one retrospective analysis of 15,532 cancer patients, 1,406 of them were given dexrazoxane; of these children, the rate of secondary acute myeloid leukemia (AML) was 0.21% in those with dexrazoxane and 0.55% in those without. The association between etoposide and secondary AML was found, but again, no association between dexrazoxane and secondary malignancy was seen [13]. From 2017, Committee for Medicinal Products for Human Use issued a change in the contraindication to dexrazoxane to only include children 0-18 years old who are receiving a cumulative dose of less than 300 kg/m^2^ of doxorubicin or an equivalent alternative anthracycline [15].

In earlier studies, dexrazoxane-induced leukopenia had been seen, but this was short term and should only be monitored and must not delay treatment [13]. Although the dose-limiting myelotoxicity of dexrazoxane is evident, it is very similar to that caused by anthracyclines, making it hard to distinguish one from another. Other adverse effects of dexrazoxane include nausea and vomiting, mucositis, increased renal excretion of iron and zinc, and transaminitis [9]. There are currently no guidelines with regard to drug monitoring; however, since the drug can lead to elevated liver enzymes, monitoring of liver function tests would be recommended during therapy.

Currently, dexrazoxane is classified as a category D drug by the FDA. In a study involving pregnant mice and rabbits, dexrazoxane leads to toxicities seen in the embryos such as malformations as well as further toxicities involving the mother itself. Some animal-based studies showed possible risks for infertility in males, evidenced by cases of testicular atrophy in dogs and rats which were attributed to dexrazoxane use. The dogs in these studies were administered doses of dexrazoxane equal to that of humans [9].

Efficacy and safety profile of dexrazoxane

The efficacy of dexrazoxane has been in question with some discrepancies noted. The Children's Oncology Group (COG) trial for de novo AML, AAML1031, found that in AML patients, a four-year risk of developing grade 2 LVSD was reduced by 45% while using dexrazoxane, demonstrating higher reduction rates with higher grades of cardiotoxicity. It was also found that five-year survival (49.0%-45.1%) and overall survival rates (65.0%-61.9%) are slightly higher with dexrazoxane use; however, the results were not statistically significant. However, the same study stated that the incidence of relapse and mortality in combined COG randomized trials of dexrazoxane showed no significant difference with or without dexrazoxane, and treatment-related mortality was lower when using dexrazoxane (p = 0.024) [6]. Similarly, a study conducted by Lipshultz et al. stated that among children treated with anthracyclines, dexrazoxane decreased clinical cardiotoxicity (p = 0.001) as well as subclinical cardiotoxicity (p <0.001) [13].

It is important to consider factors that may distort the efficacy of dexrazoxane such as gender, for example. A study conducted by Cadeddu Dessalvi et al. suggested that the risk for cardiotoxicity secondary to anthracyclines may be increased in pre-pubertal as well as adolescent females [16]. This may be due to the influence of the degree of expression of mitochondria-related oxidative genes or an ineffectual protective effect of sex hormones against the damage caused by anthracyclines in cardiac cells. One of the key factors that play into consideration with regard to the efficacy is the dose administered. A case-control study by Kim et al. in 2019 [14] showed that the cardiac event-free survival rate in patients who received more than 400 mg/m^2^ of anthracyclines, when assessing cumulative anthracycline doses, was higher in the group that received dexrazoxane.

There is no doubt that dexrazoxane has enabled patients who received anthracyclines as a treatment to have better cardiac outcomes in long-term survival. A literature review conducted by Reichardt et al. in 2018 [15] demonstrated that echocardiographic parameters such as left ventricular fraction shortening z-scores, left ventricular wall thickness, and left ventricular thickness-to-dimension z-score all had improved outcomes compared to patients who did not receive dexrazoxane. A key emphasis was based on the dose-dependent nature in which cardiotoxicity occurs; however, dexrazoxane had shown cardioprotective effects in cumulative doses up to 300 mg/m^2^; however, no statistical difference was seen in doses of 400 mg/m^2^ or above.

Although the potential for cardioprotection is significant with dexrazoxane, the safety profile is important to investigate concurrently. No significant differences in rates of infection and toxicity to the blood and central nervous system (CNS) were seen; however, rates of mucositis were found to be lower in patients taking dexrazoxane [15]. With regard to hematological toxicity and potential to develop a hematological malignancy, a lower rate of potential to develop acute myeloid leukemia (AML) was seen in patients taking dexrazoxane; however, in this study by Seif et al., no statistically significant risk was seen in developing AML by administering dexrazoxane [17]. Similarly, a study conducted by Asselin et al. in 2016 [18] had similar findings to the study conducted by Seif et al. with no differences in rates of hematological toxicity, infection, or CNS toxicity and also found lower rates of mucositis in patients receiving dexrazoxane. No evidence of an increased risk of secondary malignancy was found in patients taking dexrazoxane.

Common side effects to consider in dexrazoxane include myelosuppression, nausea, vomiting, hair loss, mucositis, transaminitis, injection site reactions, and a sporadic incidence of the development of secondary malignancies [9]. Although dexrazoxane has shown that the cardioprotective ability is substantial with this medication, it is imperative to consider the implications on the overall morbidity and mortality of patients receiving this medication. In a study conducted by Chow et al. in 2022 [19], a long-term follow-up with a median follow-up length of 18.6 years, all-cause mortality rates, and relapse rates were similar in patients who received and did not receive dexrazoxane. Although cardiomyopathy rates were lower in patients receiving dexrazoxane, no statistical difference was seen (p = 0.35); however, patients not receiving dexrazoxane did have a statistically significant risk of cardiovascular risk factors (p=0.02). No patient was known to require a heart transplant, even with anthracycline doses beyond 600 mg/m^2^.

Discussion

Anthracycline medications have been used in multiple pediatric oncological diagnoses such as neuroblastoma, Wilms' tumor, osteosarcoma, and Ewing’s sarcoma with increasing success and increase in survival rates [20]. A major concern with the use of anthracycline medications such as daunorubicin and doxorubicin is the complication of cardiotoxicity. Risk factors that have been attributed include younger ages, female gender, increased dosages, and concurrent use of radiotherapy [20]. It is important to weigh the risks and benefits of using this medication, and this is where the use of dexrazoxane plays a role in determining the consistent use of anthracyclines in a safer manner.

Dexrazoxane has been a revolutionary medication that has aided pediatric oncology patients receiving anthracycline medications to have significantly improved morbidity and mortality outcomes, especially with cardiovascular outcomes. With dexrazoxane being the only FDA-approved medication [21] as a cardioprotective agent in patients receiving anthracyclines, a substantial amount of research has been done with regard to the efficacy, safety, and clinical usability of this medication. With an overall reduction of 60% to 80% in the risk of clinical and subclinical heart failure while maintaining the appropriate anti-tumor activity of anthracyclines and having minimal side effects, dexrazoxane is a medication that has helped save the lives of patients. However, it is imperative to highlight the need for further studies in the pediatric population due to the limited number of randomized control trials. However, with the available existing data, the outcomes of the disease in the pediatric population did not change when using dexrazoxane and patients benefited from having a reduced risk of cardiac adverse events from anthracyclines. More research, however, is required to substantiate this claim. An extensive study by Zhang et al. in 2016 [22] highlighted the medications that have been or currently were being investigated, and the only medication that was efficacious was noted to be dexrazoxane. From the points highlighted in this study, it is suggestive that dexrazoxane has high clinical applicability in patients receiving anthracycline medications with a good safety profile and efficacy; however, further research not only into dexrazoxane but other medications are required to establish evidence-based efficacy.

## Conclusions

Dexrazoxane is a pivotal medication established in the care of pediatric oncology patients receiving anthracycline medications that have reduced the cardiovascular toxicity burden significantly and thereby improving morbidity and mortality outcomes in these patients. A significant amount of research is still however required within the pediatric population with regard to dexrazoxane and future medications that may be approved for use in preventing anthracycline-induced cardiotoxicity.
